# Correlation between early computed tomography findings and neurological outcome in pediatric traumatic brain injury patients

**DOI:** 10.1007/s10072-024-07511-x

**Published:** 2024-04-15

**Authors:** Süleyman Şahin, Edin Botan, Emrah Gün, Merve Feyza Yüksel, Nurşah Yeniay Süt, Ayşe Tuğba Kartal, Anar Gurbanov, Fevzi Kahveci, Hasan Özen, Merve Havan, Miraç Yıldırım, Seda Kaynak Şahap, Ömer Bektaş, Serap Teber, Suat Fitoz, Tanıl Kendirli

**Affiliations:** 1 Department of Pediatric Neurology, Ankara University Medical School, Çocuk Nöroloji Bilim Dalı, Ankara Üniversitesi Tıp Fakültesi Çocuk Sağlığı Ve Hastalıkları A.B.D. Cebeci, Ankara, Turkey; 2 Department of Pediatric Intensive Care Unit, Ankara University Medical School, Çocuk Yoğun Bakım Bilim Dalı, Ankara Üniversitesi Tıp Fakültesi Çocuk Sağlığı Ve Hastalıkları A.B.D. Cebeci, Ankara, Turkey; 3 Department of Pediatric Radiology, Ankara University Medical School, Çocuk Radyoloji Bilim Dalı, Ankara Üniversitesi Tıp Fakültesi Çocuk Sağlığı Ve Hastalıkları A.B.D. Cebeci, Ankara, Turkey

**Keywords:** Trauma, Brain injury, Neurologic outcome, Children, Intensive care, Computed tomography

## Abstract

Traumatic brain injury (TBI) is a leading cause of morbidity and mortality in children. Head computed tomography (CT) is frequently utilized for evaluating trauma-related characteristics, selecting treatment options, and monitoring complications in the early stages. This study assessed the relationship between cranial CT findings and early and late neurological outcomes in pediatric TBI patients admitted to the pediatric intensive care unit (PICU). The study included children aged 1 month to 18 years who were admitted to the PICU due to TBI between 2014 and 2020. Sociodemographic data, clinical characteristics, and cranial CT findings were analyzed. Patients were categorized based on their Glasgow Coma Scale (GCS) score. Of the 129 patients, 83 (64%) were male, and 46 (36%) were female, with a mean age of 6.8 years. Falls (*n* = 51, 39.5%) and in-vehicle traffic accidents (*n* = 35, 27.1%) were the most common trauma types observed. Normal brain imaging findings were found in 62.7% of the patients, while 37.3% exhibited intracranial pathology. Hemorrhage was the most frequent CT finding. Severe TBI (*n* = 26, *p* = 0.032) and mortality (*n* = 9, *p* = 0.017) were more prevalent in traffic accidents. The overall mortality rate in the study population was 10.1%. In children with TBI, cranial CT imaging serves as an essential initial method for patients with neurological manifestations. Particularly, a GCS score of ≤ 8, multiple hemorrhages, diffuse cerebral edema, and intraventricular bleeding are associated with sequelae and mortality.

## Introduction

Traumatic brain injury (TBI) is one of the most prevalent indications of hospitalization in children. It has a substantial association with morbidity and death [1]. Although TBI incidence varies by region [2], past studies have found that children and young adults are the most commonly involved groups. Mild TBI is more common than moderate and severe TBI [3, 4]. Although the mortality rate has dropped as a result of medical improvements, the frequency of serious sequelae and vegetative states remains high [5–7]. In recent years, there has been a growing interest in TBI's short- and long-term effects on abilities such as memory, attention, behavior, adjustment problems, and education [8–10]. Patients with severe TBI are frequently intubated and given sedatives and neuromuscular blockers; thus, the initial neurological examination may be unsatisfactory [11–13]. As a result, radiological imaging is required to determine the severity of head trauma and predict the prognosis. Non-contrast head CT is the preferred examination in trauma patients since it provides rapid imaging, particularly in intubated patients, and is widely available in most healthcare facilities.

Children who have had moderate or severe TBI are more likely to develop neurocognitive disorders and behavioral problems in the long term. Neurocognitive disorders include deficits in attention, learning, memory, and executive functions [14], whereas behavioral problems include depression, anxiety, and aggression [15]. Current studies indicate that children with risk factors for intracranial pathologies (e.g., skull fracture, persistent vomiting, focal neurological deficit) are at risk for neurocognitive and behavioral outcomes following mild TBI [16, 17]. The intricacy of TBI neuropathology shows that the end outcome may vary as a result of multiple factors (for example, management, patient-related factors, and trauma severity) [18, 19]. Thus, a better understanding of the correlations between neuroimaging and functional outcomes may help to improve the prognosis of children with TBI.

CT has limited sensitivity in identifying the effects of TBI on white matter integrity [20, 21]. This indicates that the modalities of imaging may not be sufficient to predict functional outcomes. In light of this, studies have been undertaken that question the prognostic value of CT scans for the functional outcome of pediatric TBI [22–24].

We aimed to investigate the relationship between early clinical and cranial CT findings and morbidity and mortality in children admitted to the pediatric intensive care unit (PICU) with mild, moderate, and severe TBI.

## Methods

### Patient selection

This retrospective study was conducted on patients admitted to PICU with TBI between January 2014 and 2020. The data were obtained from Ankara University Faculty of Medicine Children's Hospital Pediatric Emergency Clinic and Pediatric Intensive Care Unit patient records. This study was approved by the institutional ethical committee (21 March 2022 / i04-159–22). It included assessing trauma types, epidemiological characteristics, radiological features in the early periods, treatments performed, neurological findings and procedures, and early and late neurological status. The sociodemographic data were collected from the patient's file.

The inclusion criteria were as follows and were based on European Federation of Neurological Societies guidelines:

- Age < 18 years;
  a Mild TBI {Glasgow Coma Scale (GCS) score = 13–15}: Children in the mild TBI group were required to have a history of hospital admission with a clinical diagnosis of either:
    Unclear or ambiguous accident history, continued post-traumatic amnesia, retrograde amnesia longer than 30 min, trauma above the clavicles, including clinical signs of skull fracture, severe headache, vomiting, focal neurological deficit, seizure, coagulation disorders, high-energy accidents, and intoxication with alcohol or drugs.
  b Moderate TBI (GCS score = 9–12),
  c Severe TBI (GCS score =  ≤ 8).

The exclusion criteria included a previous history of TBI, a visual disorder that precluded neurocognitive testing, and the presence of a neurological condition other than TBI, either confirmed or reported by parents, that could impair neurocognitive function. Patients who did not have any available neuroimaging studies were excluded.

The Pediatric Cerebral Performance Category (PCPC) score was used in the PICU to assess post-arrest conditions. PCPC scores are valid and reliable methods for measuring short-term morbidity in children following intensive care unit admission [25]. The total score is from 1 to 6 (1, normal; 2, mild disability; 3, moderate disability; 4, severe disability; 5, coma or vegetative state; and 6, brain death). The patient was assessed using the PCPC scoring system in months 3 and 6 after discharge from the PICU.

During observation, the presence of one of the following findings were considered to be sufficient criteria for a cranial CT scan: witnessed loss of consciousness longer than 5 min, post-traumatic amnesia longer than 5 min, abnormal numbness, vomiting ≥ 3 episodes, suspicious history of head trauma, post-traumatic epileptic seizure (without a history of epilepsy), GCS score < 14 in infants or GCS score < 15 in children aged > 1 year in initial assessment at emergency department (ED), open or depressed skull injury and suspected fontanel swelling, any finding suggestive of skull base fracture (panda eyes, rhinorrhea, and otorrhea, hemotympanum), neurological deficit, bruise, swelling or avulsion > 5 cm in infants and type of trauma (high-velocity traffic accident, fall from height > 3 m, injury with gunfire or high-speed object).

### Data collection

We conducted a retrospective review of all children (ages 1 to 18) who were admitted to our hospital (a level 3 trauma center) with a TBI diagnosis. GCS was used to determine the severity of TBI at the ED presentation. The study sample included patients who underwent a cranial CT on admission or at any time during their stay in the intensive care unit. The cranial CT findings were classified as normal, unifocal/multifocal hemorrhage, one or both hemispheric hemorrhages, local cerebral edema, diffuse cerebral edema, and intraventricular hemorrhage. The mechanism of injury was classified as an in-vehicle traffic accident, an out-of-vehicle traffic accident, a fall, a firearm-related injury, or miscellaneous. The timing of neuroimaging was intended to be similar to that reported in adult patients [26, 27]. Unaware of the clinical data, a pediatric radiologist assessed the cranial CT images.

### Statistical analysis

SPSS statistical software version 26 was used for the analyses. Categorical data was summarized using frequencies and percentages, whereas continuous data was summarized using the mean ± SD and/or median. For analysis of between-group differences in discrete variables, the chi-square test or Fisher's exact test was employed, as appropriate; for continuous variables, analysis of variance (ANOVA) was used. A p-value < 0.05 indicated statistical significance.

## Results

During the study period, 129 patients with traumatic brain injury (TBI) were examined, with 83 boys (64%), 46 girls (36%), and a mean age of 6.8 years. Falls were the most prevalent cause (39.5%), primarily affecting younger patients (mean age: 4.9 ± 4.8 years). In this group, two patients (3.9%) succumbed, four patients (7.8%) experienced sequelae, and 47 patients (92.2%) recovered completely. In-vehicle traffic accidents were the second most prevalent cause (*n* = 35), with a mean age of 7.6 ± 5.5 years. Four patients (11.4%) died, 12 (35.3%) had sequelae, and 22 (64.7%) recovered completely (Table 1).

**Table 1:** Characteristics of study population

n (%)	
Age, months	
≤ 12	13(10)
13–60	56(43.4)
> 60	60(46.6)
Female	46(35.7)
Male	83(64.3)
TBI severity	
Mild (13–15)	79(61.2)
Moderate (9–12)	13(10.1)
Severe (3–8)	37(28.7)
Type of trauma	
Fall	51(39.5)
IVTA	35(27.1)
OVTA	33(25.6)
Gunfire injury	1(0.8)
Other	9(7)
Mortality	13(10)

*IVTA* In-vehicle traffic accident, *OVTA* Out-vehicle traffic accident

Clinical assessments indicated that 64.3% of patients had no complaints. Anisocoria was observed in 10 patients (7.7%), all classified as having severe TBI. Severe TBI (*n* = 26; *p* = 0.032) and fatal outcomes (*n* = 9; *p* = 0.017) were more common in traffic accidents. The overall mortality rate was 10.1%, with a mean Pediatric Risk of Mortality (PRISM) score of 38.4 ± 12.7 in non-survivors. The PRISM score was 37.8 ± 12.2 in patients with GCS scores of 3–8 (Table 2).

**Table 2:** Injury characteristics and triage features of 129 children diagnosed as TBI

	All cases	0–1 years	1–5 years	5–18 years
	N (%)	N (%)	N (%)	N (%)
Presentation type				
Pediatric emergency department	50 (38.8)	6 (46.2)	27 (48.3)	17 (28.3)
Same city hospitals	49 (37.9)	5 (38.4)	20 (35.6)	24 (40)
Another hospital, out of the city	30 (23.3)	2 (15.4)	9 (16.1)	19 (31.7)
Trauma type				
IVTA	35 (27.1)	4 (30.8)	11 (19.6)	20 (33.3)
OVTA	33 (25.6)	1 (7.7)	11 (19.6)	21 (35)
Fall	51 (39.5)	8 (61.6)	29 (51.9)	14 (23.4)
Gunfire injury	1 (0.8)	0 (0)	0 (0)	1 (1.65)
Other	9 (7)	0 (0)	5 (8.9)	4 (6.65)
Consciousness				
Full	83 (64.4)	10 (76.9)	35 (62.5)	38 (63.3)
Confusion	11 (8.5)	0 (0)	6 (10.7)	5 (8.4)
Lethargy	3 (2.3)	0 (0)	1 (1.8)	2 (3.3)
Stupor	1 (0.8)	0 (0)	1 (1.8)	0 (0)
Coma	31 (24)	3 (23.1)	13 (23.2)	15 (25)
GCS				
3–8	37 (28.7)	3 (23.1)	15 (26.8)	19 (31.7)
9–12	13 (10.1)	1 (7.7)	6 (10.7)	6 (10)
13–15	79 (61.2)	9 (69.2)	35 (62.5)	35 (58.3)
Anisocoria	10 (7.8)	0 (0)	5 (8.3)	5 (8.9)
PRISM (mean)	7.622	4.077	7.857	8.190

GCS scores were 3–8 in 28.7% (*n* = 37), 9–12 in 10.1% (*n* = 13), and 13–15 in 61.2% (*n* = 79). Full recovery was common in patients with a GCS score of 13–15, and this group had the highest number of Category 1 patients, according to PCPC. Favorable disease progression was significantly higher in patients with higher GCS scores (*p* < 0.05). Death or poor outcomes were more common in the low GCS group (*p* < 0.001).

Cranial CT examinations were conducted for all patients, with 62.7% showing normal findings (Table 3). Abnormalities included unifocal hemorrhage (20.1%), multifocal hemorrhage (17%), and diffuse cerebral edema (9), with 7 having severe TBI. Pathological cranial CT features were not associated with extracranial injury findings in 52.1% of cases. Patients with normal initial CT findings (*n* = 81) had low mortality (3.7%), while those with abnormal findings (*n* = 48) had higher mortality (20.7%). Different types of hemorrhages have varying mortality and sequelae rates.

**Table 3:** Percentage of abnormal CT findings in children underwent CT scan, according to age

Age, months	Number of abnormal CT scan/Number of patients underwent CT scan	%
≤ 12	2/13	15.3
13–60	22/56	39.3
> 60	24/60	40
Total	48/129	37.2

Of the 129 patients, 66 (51.1%) received supportive care alone, while 63 received medical therapy. Six required surgery and mechanical ventilation were performed in 31 children (24%). The overall mortality rate was 10.0%. Of survivors (*n* = 117), 96 patients (79%) were discharged without permanent sequelae, 21 (16.2%) were discharged with sequelae, and 9 (6.9%) developed epilepsy.

The mean PICU stay was longer for boys, and patients with pathological CT findings had extended ICU stays. The presence of anisocoria was significantly correlated with sequelae and death. GCS scores ranged from 3 to 8 in all patients with anisocoria (*n* = 10), and 90% had abnormal CT scans. Anisocoria was a notable poor prognostic factor (Tables 4 and 5).

**Table 4:** Correlation between treatment choices and GCS score and outcome

	All cases	GCS			Epilepsy	Sequelae	Death
Treatment	n	3–8 points (n/%)	9–12 points (n/%)	12–15 points (n/%)	n	n	n
3% NaCl	52	25(48)	8(15.4)	19(36.6)	4	11	10
Mannitol	1	1(100)	–	–	–	1	–
Dexamethasone	8	6(75)	1(12.5)	1(12.5)	1	3	2
Craniectomy	6	4(66.6)	–	2(33.4)	2	3	1
Antiepileptic	51	22(43.1)	5(9.8)	24(47.1)	7	7	7

**Table 5:** Correlation of extra-cranial injury with GCS and morbidity

	GCS			Sequelae	Death	Tracheostomy	Length of PICU stay
Extra-cranial injury	3–8 n(%)	9–12 n(%)	12–15 n(/%)	n	n	n	Day, mean
Yes	22(59.5)	7(53.8)	41(51.9)	13	7	5	12.1
No	15(40.5)	6(46.2)	38(48.1)	8	6	–	5.5

All patients were admitted to the intensive care unit after initial therapeutic measures by the ED. Hypnotic agents were administered to facilitate mechanical ventilation. Hypertonic saline, dexamethasone, and/or mannitol were used for cerebral edema (Table 4). No adverse events related to treatment were observed. Statistical analysis showed that anisocoria and extra-cranial injuries were associated with various clinical outcomes (Table 5).

Patients involved in out-of-vehicle traffic accidents had the worst PCPC category and the second-most common pathological CT findings (Table 6).

**Table 6:** Correlation of PCPC scale with coma severity, extra-cranial injury and CT findings

PCPC on month 3		PCPC on month 6
TBI (*p* < 0.001)		
Mild (GCS 13–15 points)	1.0 ± 0	1.0 ± 0
Moderate (GCS 9–12 points)	1.2 ± 0.8	1.2 ± 0.8
Severe (GCS 3–8 points)	2.3 ± 1.1	2.3 ± 1.1
Extra-cranial injury		
Yes	1.5 ± 1	1.5 ± 1
No	1.3 ± 0.7	1.3 ± 0.7
CT finding (*p* < 0.001)		
Yes	1.9 ± 1.1	1.9 ± 1.1
No	1.1 ± 0.6	1.1 ± 0.6
EEG finding (*p* < 0.001)		
Yes	2.1 ± 1.2	2.1 ± 1.2
No	1.3 ± 0.7	1.2 ± 0.7

PCPC scores did not change at 90 and 180 days for patients with severe, moderate, and mild TBI at admission. The mean PCPC score was 0.4 units higher in patients with extra-cranial injury than those without (Table 6).

A comparison of PCPC scores at 90 and 180 days revealed significant differences between patients with and without CT findings. Pathological results, including various abnormalities, were observed in 73.9% of the patients who underwent EEG.

A comparison of GCS scores at admission and discharge revealed improvement in some patients. Extra-cranial injuries were associated with various clinical outcomes.

Patients involved in out-of-vehicle traffic accidents had the worst PCPC category and the second-most common pathological CT findings (Table 6).

It was observed that mortality increased with an increasing Rotterdam score (RS). RS was significantly higher in patients who died at the end of the follow-up period than in those who survived (Table 7). The Rotterdam scoring system had good discriminatory power in predicting early mortality. In our study, the mean RS was obtained as 2.4 ± 0.9. This rate was 3.7 ± 0.5 in deceased patients and 2.2 ± 0.9 in surviving patients (Table 8). Mortality was associated with increased RS, as shown in figure x. A moderate and positive relationship was found between the patients' RS and PCPC scores at 3 months (*r* = 0.544, *p* < 0.01) and PCPC scores at 6 months (*r* = 0.518). As patients' RS increased, PCPC measurements increased in the 3rd month and the 6th month (Table 9). The non-parametric Spearman-Brown correlation method was used to determine the relationship between PCPC measurements and RS at 3 and 6 months. PCPC measurements at the 3rd and 6th months are an ordinal variable, and this non-parametric method is used in the relationship between ordinal type variables.

**Table 7:** Rotterdam Scoring System

CT scan feature	Score
Basal cisterns	
• Normal	0
• Compressed	1
• Absent	2
Midline shift	
• No shift or shift ≤ 5 mm	0
• Shift > 5 mm	1
Epidural mass lesion	
• Present	0
• Absent	1
IVH or SAH	
• Absent	0
• Present	1
Sum score	+ 1

*CT* computed tomography, *IVH* intraventricular hemorrhage, *SAH* subarachnoid hemorrhage

**Table 8:** Relation between RS and mortality

Groups	*N*	Mean ± sd	*p*
Mortality			
Ex	13	3,7 ± 0,5	< 0,001*
Alive	116	2,2 ± 0,9

**Table 9:** Corelation table

	1	2	3
1.3rd month PCPC	1		
2.6th month PCPC	,982**	1	
3.RS	,544**	,518**	1

^**^p < .01

A comparison of PCPC scores at the 90th and 180th days revealed significant differences between patients who had CT findings and those who didn't. Pathological findings, including various abnormalities, were observed in 73.9% of patients with an EEG.

Anti-epileptic agent treatment was initiated in 39.5% of patients, and 28 patients discontinued treatment before discharge. Patients who were discharged after receiving anti-epileptic treatment had a higher rate of sequelae and epilepsy.

## Discussion

The initial phase of TBI cases admitted to the PICU is marked by potential morbidity and mortality as a result of trauma and hospitalization [28]. Following discharge, these individuals may grapple with persistent neurological and physical challenges with enduring consequences [28]. Along with the discernible neurophysical aftereffects, neuropsychological sequelae can have a significant impact on vital developmental functions such as learning, emotional comprehension, and social functioning. As a result, consistent follow-up is critical in addressing the potential appearance of new-onset seizures and providing adequate treatment for physical, behavioral, and cognitive deficits [29]. Consequently, rehabilitating long-term sequelae through preemptive measures, early detection, and efficacious TBI management emerges as highly important for ensuring affected children's survival and holistic quality of life.

TBI ranks as the predominant cause of childhood mortality [30]. Notable differentials manifest in injury mechanisms, trauma categories, and PICU management approaches when juxtaposed against the adult demographic [31]. In our investigation, the mean patient age tallied at 6.8 years, in contrast to 5.5 years in Chaitanya et al.'s study [32], 5.5 years in the Langlois J et al. research [33], and 4 years in Pranshu B et al.’s study [31]. Moreover, our study showcased a male-to-female ratio of 1.8:1, which concurs with Chaitanya et al.'s [32] and Satapathy M et al.’s ratios [34]. However, in the pediatric head trauma series, Sambasivan [35] reported a gender-agnostic incidence. Chiaretti et al. speculated that the higher male TBI incidence could be attributed to greater head circumference, enhanced muscle mass, and increased physical activity relative to females [36].

Multiple investigations have spotlighted the prevalent injury mechanism in pediatric TBI as falling from heights, followed by traffic accidents [31, 37–40]. Shaylan and Neil's study [41] underscored traffic accidents as the foremost contributor, accounting for 68% of the 1,298 pediatric TBI cases recorded between 2000 and 2015. In our study, traffic accidents emerged as the primary TBI causative factor [34, 37, 38]. However, falls assumed the forefront upon categorizing traffic accidents into in-vehicle and out-of-vehicle incidents. This outcome concurs with earlier studies, where traffic accident-related TBIs were predominantly attributed to out-of-vehicle scenarios [30, 37].

Our study revealed that children with mild TBI constituted 61.2% of the subject pool. Comparable proportions were noted as 60.5% by Chaitanya et al. [32], 65.3% by Kupperman N et al. [42], 68.7% by Satapathy M et al. [34], and 70% by Gururaj G et al. [29]. Regarding CT findings, unifocal hemorrhage was the most recurrent discovery, trailed by multifocal hemorrhage, localized brain edema, diffuse cerebral edema, and intraventricular hemorrhage in our study. Conversely, the literature reveals fluctuations in findings; for instance, Chaitanya et al. [32] identified skull fractures as the primary finding, while Nath et al. [43] reported contusions. A similar trend was observed by Pranshu et al. [31]. Further, Satapathy et al.'s study [34] highlighted extra-dural hematoma as the prevailing observation. Our study additionally observed that CT scans returned expected results in 62.8% of cases, substantially surpassing the rates of 13.4% in Kumar A et al.'s research [38], 26% in Chaitanya et al.'s study [32], and 16.3% in Satapathy M et al.'s investigation [34].

Neurosurgical intervention was warranted in 4.6% of cases, a lower occurrence compared to Chaitanya et al.'s [32] 10.5%, Satapathy M et al.'s [34] 20%, and Bahloul et al.'s [44] 25%. Various sequelae may follow TBI in children, encompassing epilepsy, muscle weakness, blindness, hydrocephalus, and memory loss. Our study documented sequelae in 16.2% of patients. Commensurate rates were reported as 14% by Ji-Yao Jiang et al. [45], 15% by Bahloul M et al. [44], 21% by Chaitanya et al. [32], and 9% by Satapathy M et al. [34]. Most treatment approaches in our study were conservative, given the prevalence of mild TBI cases (GCS score 13–15). Substantial recovery was observed in 74.4% of cases, while the overall mortality rate was 10%.

The GCS score at admission exhibited a direct correlation with the severity of head trauma, subsequently influencing morbidity and mortality. This finding suggests improved outcomes compared to prior studies [31, 34, 37]. However, the study's time frame limited our insights into long-term neurocognitive outcomes post-discharge.

Clinical assessment revealed varying levels of consciousness, with a significant percentage showing no complaints. Anisocoria, present in severe TBI cases, emerged as a notable indicator of poor prognosis. Concurrent extracranial injuries were prevalent and associated with extended PICU stays, prolonged mechanical ventilation, tracheostomy at discharge, and longer hospital stays.

In this study, 25 of 59 children with no obvious external injury had a significant intracranial injury on a CT scan; therefore, the absence of external injury does not exclude TBI.

Unifocal bleeding was the most common CT scan finding, followed by multifocal hemorrhage and cerebral edema, in this study, whereas Mahapatra et al. [38] report contusion as the most common; a similar observation has been reported by Pranshu et al. [31]. Extradural hemorrhage was the most common finding in Satapathy et al. 's study.[34]. Normal CT scan findings were seen in 62,8% of cases in our study, higher than 16.3% in the study by Satapathy et al. [34] and 13.48% in the study by Mahapatra et al. [38].

Anisocoria, abnormal EEG findings, and treatment were associated with the PCPC score, indicating their importance in predicting outcomes. The analysis of the patients without and with CT findings at follow-up days showed significant differences in PCPC scores.

We found that RS on CT was directly related to the outcomes of children admitted to our PICU with TBI. Various clinical parameters, including age, mechanism of injury, initial Glasgow coma scale, hypoxia, hypotension, and abnormal pupillary response, have been identified to predict the long-term outcomes of patients with TBI 8. Also, recently, initial brain CT scans in the emergency department have been increasingly used to determine TBI patients' outcomes. It is used and defined as a step that should be included in risk estimation [46, 47].

There is strong evidence in the adult TBI literature that RS is an independent prognostic indicator. RS is one such measurement that combines CT scan findings to describe the heterogeneity of lesions and their associated prognosis [48, 49].

There is a lack of knowledge regarding the using RS in pediatric TBI. Liesemer and colleagues found that RS directly associated with mortality in a large pediatric cohort of more than 600 pediatric patients [50].

## Conclusions

In conclusion, the findings from this study's cranial CT scans highlight the importance of initial imaging assessments in predicting patient outcomes following TBI. A significant portion of patients (62.7%) exhibited normal brain CT findings, highlighting the potential challenges of identifying TBI-related abnormalities solely through imaging. However, distinct patterns occurred among patients with abnormal CT findings (37.3%). Notably, unifocal hemorrhage was associated with a mortality rate of 15.4%, but multifocal hemorrhage had a mortality rate of 27.3%. Diffuse cerebral edema was especially concerning as it resulted in a greater fatality rate, whereas focal cerebral edema also had a notable impact on patient outcomes. It is noteworthy that the presence of pathological cranial CT features did not necessarily correlate with extra-cranial injury findings in more than half of the cases.

There is strong evidence in the adult TBI literature that RS is an independent prognostic indicator. RS is one such measurement that combines CT scan findings to describe the heterogeneity of lesions and their associated prognosis. This study gains importance with its evidence regarding the positive relationship between RS and mortality and long-term outcomes (PCPC) in the pediatric patient group.

Furthermore, the analysis of patient outcomes found significant trends. Among individuals with normal initial CT findings, only a small percentage died or recovered with sequelae. Patients with abnormal cranial CT findings, on the other hand, had higher mortality rates, and a large proportion suffered from sequelae. These findings highlight the complexities of TBI cases and the significance of a precise diagnosis for effective treatment and care strategies. The disparities in outcomes based on the type and degree of intracranial anomalies underline the importance of targeted medical interventions and comprehensive post-TBI management. Finally, these insights contribute to a better understanding of the prognostic implications of various cranial CT findings, allowing clinicians to make more informed decisions that improve patient outcomes and quality of life following a traumatic brain injury.

The results reported in the article add to the growing body of studies emphasizing the need to view TBI, particularly in moderate-to-severe cases, as a chronic disease. This recognition implies that the effects of TBI go well beyond the initial injury and healing time, affecting an individual's life in the long run. TBI can have long-term effects on a person's well-being in a various ways, including physical, cognitive, emotional, and social dimensions. Such a perspective emphasizes the critical importance of continued care, support, and management for TBI survivors in order to address the long-term challenges in multiple domains of their lives.

## Data Availability

The data supporting this study's findings are available on request from the corresponding author, [Ş.S.]. The data are not publicly available due to [restrictions, e.g., their containing information that could compromise the privacy of research participants].

## References

[1] Behrens TE, Woolrich MW, Jenkinson M et al (2003) Characterization and propagation of uncertainty in diffusion-weighted MR imaging. Magn Reson Med 50:1077–1088 14587019 10.1002/mrm.10609

[2] Keenan HT, Runyan DK, Marshall SW et al (2003) A population-based study of inflicted traumatic brain injury in young children. JAMA 290:621–626 12902365 10.1001/jama.290.5.621

[3] Numminen HJ (2011) The incidence of traumatic brain injury in an adult population-How to classify mild cases? Eur J Neurol 18:460–464 20722703 10.1111/j.1468-1331.2010.03179.x

[4] Bazarian JJ, McClung J, Cheng YT et al (2005) Emergency department management of mild traumatic brain injury in the USA. Emerg Med J 22:473–477 15983080 10.1136/emj.2004.019273 PMC1726852

[5] Andriessen TM, Horn J, Franschman G et al (2011) Epidemiology, severity classification, and outcome of moderate and severe traumatic brain injury: a prospective multicenter study. J Neurotrauma 28:2019–2031 21787177 10.1089/neu.2011.2034

[6] Brain Trauma Foundation; American Association of Neurological Surgeons, Congress of Neurological Surgeons (2007) Guidelines for the management of severe traumatic brain injury. J Neurotrauma. 24(Suppl 1):S1-106 10.1089/neu.2007.9999 17511534

[7] Kan CH, Saffari M, Khoo TH (2009) Prognostic factors of severe traumatic brain injury outcome in children aged 2–16 years at a major neurosurgical referral centre. Malays J Med Sci 16:25–33 22135509 PMC3216137

[8] Catroppa C, Anderson VA, Muscara F et al (2009) Long-term outcome and predictors following paediatric traumatic brain injury. Neuropsychol Rehabil 19:716–732 19306233 10.1080/09602010902732868

[9] Muscara F, Catroppa C, Eren S et al (2009) The impact of injury severity on long-term social outcome following paediatric traumatic brain injury. Neuropsychol Rehabil 19:541–561 18839384 10.1080/09602010802365223

[10] Fletcher JM, Levin HS, Lachar D et al (1996) Behavioral outcomes after pediatric closed head injury: relationships with age, severity, and lesion size. J Child Neurol 11:283–290 8807417 10.1177/088307389601100404

[11] Adelson PD, Bratton SL, Carney NA, et al. American Association for Surgery of Trauma; Child Neurology Society; International Society for Pediatric Neurosurgery; International Trauma Anesthesia and Critical Care Society; Society of Critical Care Medicine; World Federation of Pediatric Intensive and Critical Care Societies (2003) Guidelines for the acute medical management of severe traumatic brain injury in infants, children, and adolescents. Chapter 3. Prehospital airway management. Pediatr Crit Care Med 4 Suppl 1:S9–S11 12847339

[12] Badjatia N, Carney N, Crocco TJ, et al. Brain Trauma Foundation; Center for Guidelines Management. Guidelines for prehospital management of traumatic brain injury. Prehosp Emerg Care. 2008;12 Suppl 1:S1–52 10.1080/10903120701732052 18203044

[13] Kochanek PM, Carney N, Adelson PD et al (2012) Guidelines for the acute medical management of severe traumatic brain injury in infants, children, and adolescents. Pediatr Crit Care Med 13:S1-82 22217782 10.1097/PCC.0b013e31823f435c

[14] Babikian T, Asarnow R (2009) Neurocognitive outcomes and recovery after pediatric TBI: meta-analytic review of the literature. Neuropsychology 23:283–296 19413443 10.1037/a0015268 PMC4064005

[15] Li L, Liu J (2013) The effect of pediatric traumatic brain injury on behavioral outcomes: a systematic review. Dev Med Child Neurol 55:37–45 22998525 10.1111/j.1469-8749.2012.04414.x PMC3593091

[16] Königs M, Heij HA, Van der Sluijs JA et al (2015) Pediatric traumatic brain injury and attention deficit. Pediatrics 136:534–541 26240208 10.1542/peds.2015-0437

[17] Königs M, Weeda WD, Van Heurn LWE et al (2015) Impaired visual integration in children with traumatic brain injury: an observational study. PLoS ONE 10(12):e0144395 26637182 10.1371/journal.pone.0144395 PMC4670090

[18] Bigler ED, Abildskov T, Petrie J (2013) Heterogeneity of brain lesions in pediatric traumatic brain injury. Neuropsychology 4:438–451 10.1037/a0032837 23876117

[19] Bigler ED, Maxwell WL (2012) Neuropathology of mild traumatic brain injury: relationship to neuroimaging findings. Brain Imaging Behav 6:108–136 22434552 10.1007/s11682-011-9145-0

[20] Mittl RL, Grossman RI, Hiehle JF et al (1994) Prevalence of MR evidence of diffuse axonal injury in patients with mild head injury and normal head CT findings. Am J Neuroradiol 15:1583–1589 7985582 PMC8334423

[21] Sigmund GA, Tong KA, Nickerson JP et al (2007) Multimodality comparison of neuroimaging in pediatric traumatic brain injury. Pediatr Neurol 36:217–226 17437903 10.1016/j.pediatrneurol.2007.01.003

[22] Blackman JA, Rice SA, Matsumoto JA et al (2003) Brain imaging as a predictor of early functional outcome following traumatic brain injury in children, adolescents, and young adults. J Head Trauma Rehabil 18:493–503 14707879 10.1097/00001199-200311000-00003

[23] Gerlach R, Dittrich S, Schneider W et al (2009) Traumatic epidural hematomas in children and adolescents: outcome analysis in 39 consecutive unselected cases. Pediatr Emerg Care 25:164–169 19262419 10.1097/PEC.0b013e31819a8966

[24] Mendelsohn D, Levin HS, Harward H et al (1992) Corpus Callosum lesions after closed head injury in children: MRI, clinical features and outcome. Neuroradiology 34:384–388 1407515 10.1007/BF00596495

[25] Debra Henry Fiser (1992) Assessing the outcome of pediatric intensive care. J Pediatr 121:68–74 1625096 10.1016/s0022-3476(05)82544-2

[26] Lee H, Wintermark M, Gean AD et al (2008) Focal lesions in acute mild traumatic brain injury and neurocognitive outcome: CT versus 3T MRI. J Neurotrauma 25:1049–1056 18707244 10.1089/neu.2008.0566

[27] Yuh EL, Mukherjee P, Lingsma HF, et al. TRACK-TBI Investigators. Magnetic resonance imaging improves 3-month outcome prediction in mild traumatic brain injury. Ann Neurol. 2013;73:224–235 10.1002/ana.23783 PMC4060890 23224915

[28] Guilliams K, Wainwright MS (2016) Pathophysiology and management of moderate and severe traumatic brain injury in children. J Child Neurol 31:35–45 25512361 10.1177/0883073814562626

[29] Gururaj G (2002) Epidemiology of traumatic brain injuries: Indian scenario. Neurol Res 24:24–28 11783750 10.1179/016164102101199503

[30] Luerssen T, Klauber M, Marshall L (1988) Outcome from head injury related to patient’s age. A longitudinal prospective study of adult and pediatric head injury. J Neurosurg 68(409):416 10.3171/jns.1988.68.3.0409 3343613

[31] Pranshu B, Rahul S, Bhanu P et al (2011) Pediatric head injury: an epidemiological study. J Pediatr Neurosci. 6:97–98 21977109 10.4103/1817-1745.84428 PMC3173936

[32] Chaitanya K, Addanki A, Karambelkar R et al (2018) Traumatic brain injury in Indian children. Childs Nerv Syst 34:1119–1123 29594463 10.1007/s00381-018-3784-z

[33] Langlois J, Rutland-Brown W, Thomas K (2005) The incidence of traumatic brain injury among children in the United States. J Head Trauma Rehabil 20:229–238 15908823 10.1097/00001199-200505000-00006

[34] Satapathy MC, Dash D, Mishra SS et al (2016) Spectrum and outcome of traumatic brain injury in children <15 years: a tertiary level experience in India. Int J Crit Illn Inj Sci 6:16–20 27051617 10.4103/2229-5151.177359 PMC4795356

[35] Sambasivan M (1995) Epidemiology-pediatric head injuries. Indian J Neurol 43:57–58

[36] Chiaretti A, De Benedictis R, Della Corte F et al (2001) The impact of initial management on the outcome of children with severe head injury. Childs Nerv Syst 18:54–60 11935245 10.1007/s00381-001-0533-4

[37] Tabish A, Lone N, Afzal W et al (2006) The incidence and severity of injury in children hospitalised for traumatic brain injury in Kashmir. Injury 37:410–415 16569405 10.1016/j.injury.2006.01.039

[38] Kumar R, Mahapatra AK (2009) The changing “epidemiology” of pediatric head injury and its impact on the daily clinical practice. Childs Nerv Syst 25:813–823 19212766 10.1007/s00381-009-0820-z

[39] McKinlay A, Grace R, Horwood L et al (2006) Prevalence of traumatic brain injury among children, adolescents and young adults: prospective evidence from a birth cohort. Brain Inj 22:175–181 10.1080/02699050801888824 18240046

[40] Bayreuther J, Wagener S, Woodford M et al (2009) Paediatric trauma: injury pattern and mortality in the UK. Arch Dis Child Educ Pract 94:37–41 10.1136/adc.2007.132787 19304898

[41] Govind SK, Merritt NH (2018) A 15 year cohort review of in-hospital pediatric trauma center mortality: a catalyst for injury prevention programming. Am J Surg 216:567–572 29530278 10.1016/j.amjsurg.2018.03.001

[42] Kuppermann N, Holmes J, Dayan P et al (2009) Identification of children at very low risk of clinically-important brain injuries after head trauma: a prospective cohort study. Lancet 374:1160–1170 19758692 10.1016/S0140-6736(09)61558-0

[43] Nath HD, Tandon V, Mahapatra AK et al (2015) Outcome of pediatric head injury patients admitted as unknown at a level-i apex trauma centre. Asian J Neurosurg 10:149–152 26396599 10.4103/1793-5482.161183 PMC4553724

[44] Bahloul M, Chelly H, Chaari A et al (2011) Isolated traumatic head injury in children: analysis of 276 observations. J Emerg Trauma Shock 4:29–36 21633564 10.4103/0974-2700.76831 PMC3097575

[45] Jiang J, Gao G, Li W et al (2002) Early indicators of prognosis in 846 cases of severe traumatic brain injury. J Neurotrauma 19:869–874 12184856 10.1089/08977150260190456

[46] Nelson DW, Nyström H, MacCallum RM, Thornquist B, Lilja A, Bellander BM et al (2010) Extended analysis of early computed tomography scans of traumatic brain injured patients and relations to outcome. J Neurotrauma 27(1):51–64 19698072 10.1089/neu.2009.0986

[47] Mata-Mbemba D, Mugikura S, Nakagawa A, Murata T, Ishii K, Li L et al (2014) Early CT findings to predict early death in patients with traumatic brain injury: Marshall and Rotterdam CT scoring systems compared in the major academic tertiary care hospital in northeastern Japan. Acad Radiol 21(5):605–611 24703472 10.1016/j.acra.2014.01.017

[48] Maas AI, Hukkelhoven CW, Marshall LF, Steyerberg EW (2005) Prediction of outcome in traumatic brain injury with computed tomographic characteristics: a comparison between the computed tomographic classification and combinations of computed tomographic predictors. Neurosurgery 57(6):1173–1182 16331165 10.1227/01.neu.0000186013.63046.6b

[49] Talari HR, Fakharian E, Mousavi N, Abedzadeh-Kalahroudi M, Akbari H, Zoghi S (2016) The Rotterdam scoring system can be used as an independent factor for predicting traumatic brain injury outcomes. World Neurosurg 87:195–199 26704195 10.1016/j.wneu.2015.11.055

[50] Liesemer K, Riva-Cambrin J, Bennett KS, Bratton SL, Tran H, Metzger RR et al (2014) Use of Rotterdam CT scores for mortality risk stratification in children with traumatic brain injury. Pediatr Crit Care Med 15(6):554–562 24751786 10.1097/PCC.0000000000000150 PMC4087067

